# The effect of spatial distances between holes and time delays between bone drillings based on examination of heat accumulation and risk of bone thermal necrosis

**DOI:** 10.1186/s12938-019-0686-6

**Published:** 2019-05-24

**Authors:** Seifollah Gholampour, Hossein Haghighi Hassanali Deh

**Affiliations:** 1 Department of Biomedical Engineering, Islamic Azad University-North Tehran Branch, P.O.B. 1651153311, Tehran, Iran; 2 Department of Manufacturing Engineering, Islamic Azad University-North Tehran Branch, Tehran, Iran

**Keywords:** Orthopedic surgery, Coolant, CO_2_, Temperature durability, Infrared thermography, Thermal necrosis

## Abstract

**Background and objective:**

This study was designed to investigate heat accumulation and bone thermal necrosis for various distances between holes and time delays between drillings.

**Methods:**

The tests were performed at three distances (6, 12, 16 mm) and three time delays: 0, 5 and 10 s. To examine the efficiency of coolants, CO_2_ coolant was also tested in addition to two common cooling modes in bone drilling.

**Results:**

The main results were the trend of temperature–time graph, maximum temperature at drilling site, temperature distribution on the surface of drilling site, temperature durability and returning time. The effect of lateral drillings on the initial hole was notable in drilling at a distance of 6 mm without cooling. This effect did not disappear even by increasing the time delay up to 10 s. The results obtained for drilling with normal saline coolant were not sufficiently acceptable due to the manual and non-uniform cooling process as well as the relative obstruction of the chips exit path. Generally, drillings with two common cooling modes, even when the distances between holes and time delays between drillings were controlled, did not yield all favorable conditions for preventing bone thermal necrosis.

**Conclusion:**

Bone drilling using CO_2_ coolant eliminates the risk of bone thermal necrosis completely even in cases that the distances between holes in plates or implants are 6 mm and there is no time delay between drillings. These results can be especially useful in emergency orthopedic surgeries and for designing the location of screw holes in implants and plates.

## Background

Bone drilling is usually required for inserting implants and fixing plates in fractured bones [1, 2]. Despite the various previous studies about biomechanical assessment in areas of biology [3–8], there are serious concerns related to development of bone drilling process during orthopedic surgeries. One of the main causes of this matter is the coefficient of thermal conductivity of the human bone that is between 0.38 and 2.3 W/mK [9]. Due to the low amount of this coefficient, heat remains at the drilling site during bone drilling and leads to local temperature increase and changes in the nature of alkaline phosphatase in bone [10, 11]. This provides the conditions for thermal necrosis, death of bone cells, decrease in mechanical strength of the drilling site, possible damages to the peripheral nerves and vessels at drilling site, and post-operation complications [12–15]. Therefore, investigating the phenomenon “thermal necrosis” during bone drilling is of great importance and numerous studies have been done in this regard in the past.

In the first group of studies, the variables affecting the manner and procedure of bone drilling to reduce thermal necrosis were evaluated. Gholampour et al. examined the effect of drilling depth and direction on thermal necrosis in tibia [16]. The other studies suggested the most suitable feed rate, cooling mode and rotational speed for bone drilling in two conditions of high speed drilling, and ultrasonic-assisted drilling [17, 18]. Tai et al. proposed a suitable strategy for sequential bone drilling to minimize thermal necrosis [19]. Palmisano et al. examined the effect of drilling field and number of drillings on reduction of bone thermal necrosis [20]. Buchan et al. proposed a method for improving the safety of bone drilling process [21]. Some of the other studies evaluated the effect of biomechanical properties of screws during orthopedic surgeries [22, 23].

In the second group of studies, the effect of the drilling tool type on reduction of bone thermal necrosis was investigated. Hein et al. and Palmisano et al. compared the heat generated during drillings with industrial and orthopedic drill bits [24, 25]. Udiljak et al. and Scarano et al. examined the effect of a two-phase drill bit (step drill bit) as well as the cone-shape of the external surface of drill bit on reduction of temperature and death probability of bone cells during drilling [26, 27]. In the other studies, the effect of diameter, helix angle, point angle and 2- and 3-fluted of drill bits on reduction of bone thermal necrosis were investigated [28, 29].

Some studies also measured the temperature changes at the surface of drilling site using a thermography camera considering the importance and notable impact of temperature distribution at the surface of drilling site on occurrence of thermal necrosis. Stumm et al. proposed the thermo-mechanical coefficients and parameters affecting bone thermography [30]. Kim et al. measured the temperature distribution at the surface of drilling site during low-speed bone drilling [31].

Based on the literature, comprehensive researches have been performed regarding the optimal conditions and manner of bone drilling for minimizing thermal necrosis. Inserting implants or fixing plates requires usually drilling several holes in bone. Consequently, determining the optimal distances between holes and proper time delay between each drilling and the next one is important. However, none of the previous studies have examined the effect of heat accumulation due to change in these distances and time delays on bone thermal necrosis. The present study investigates these effects and due to the importance of coolants in reducing the risk of bone thermal necrosis during bone drilling, compares the efficacy of internal gas coolants with that of two cooling modes commonly used in operation rooms (drilling without cooling and drilling with normal saline as coolant) with the aim of improving the efficacy of orthopedic surgeries.

## Methods

The femoral diaphysis of 23 grown-ups (44% females and 46% males) was used as test specimens. These specimens were cut in dimensions of 25 mm to make testing easier and each test was repeated seven times. The specimens were stored according to the recommendation of Sedlin & Hirsch and Hillery et al. at − 20 °C in Ringer’s solution and then were tested [9, 32].

First, the effect of distance between the holes on insertion of plates and implants was investigated during drilling with the aim of minimizing bone thermal necrosis. To do this, an initial hole was created in the bone. Then lateral holes were drilled at a distance of 6 mm from the center of the initial hole so that the effect of accumulation of the generated heat due to drilling of lateral holes on the initial hole and its effect on bone thermal necrosis could be investigated. It should be noted that the three values of 6, 12 and 16 mm are the most common distances between the screw holes in a variety of implants and plates. Thus, the above experiments were repeated for distances of 12 and 16 mm between the centers of the initial and lateral holes.

The next variable in successive drillings was the time interval between the first drilling and the lateral drillings. Hence, in the second part of the experiment, the lateral holes were drilled without delay after drilling the initial hole. The experiment was then repeated for time delays of 5 and 10 s to assess the effect of time delay between drillings on reduction or raise of heat accumulation due to lateral drillings at the initial hole. It should be noted that all drillings were done at a depth of 8 mm with a rotational speed of 2000 rpm as the most optimal rotational speed for minimizing thermal necrosis [9, 16].

**Fig. 1:**
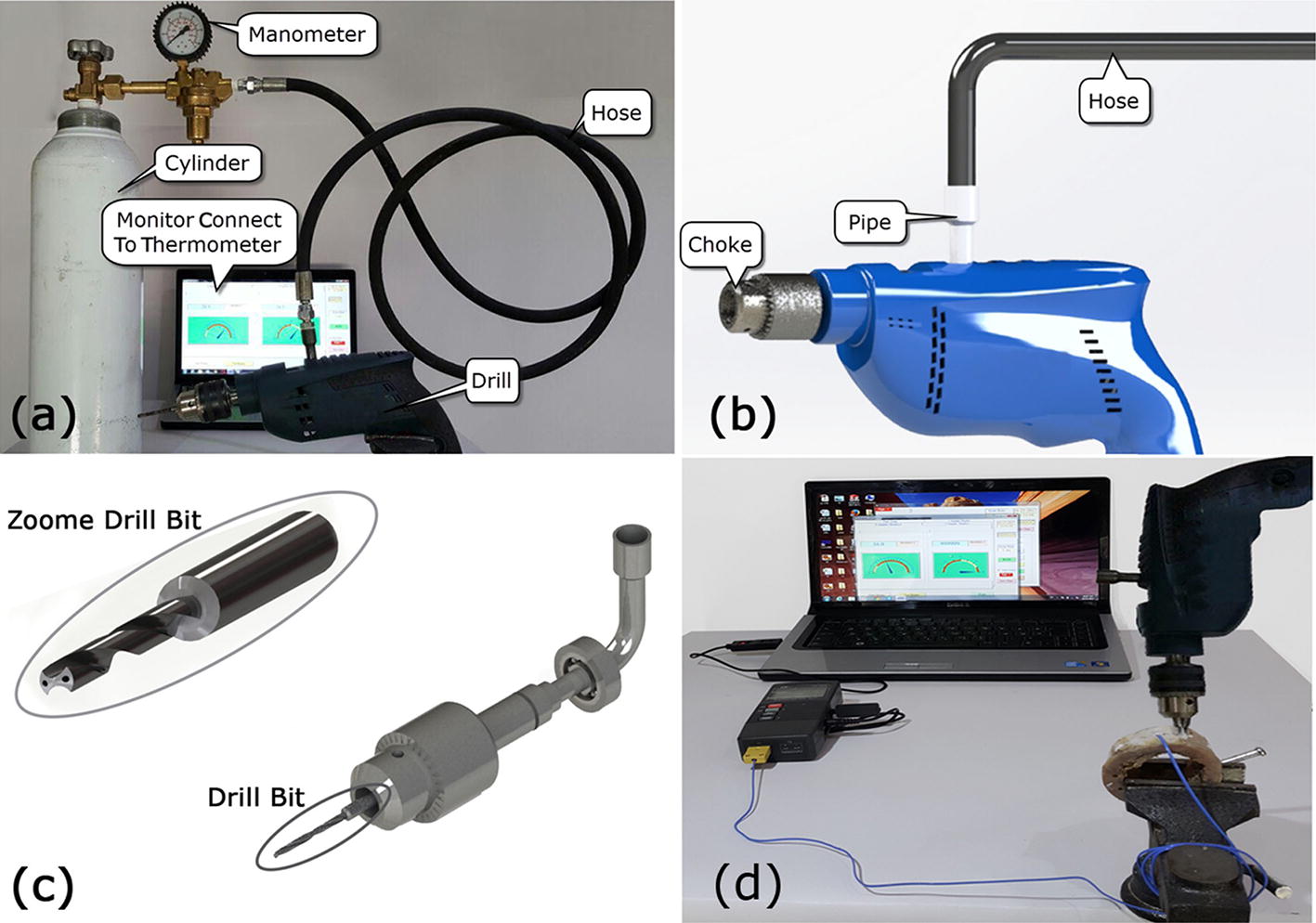
**a** Equipment of experiment, **b** schematic view of drill, **c** gas runner and internal coolant drill bit, **d** a view of Lutron thermometer

Considering the importance of coolants in reduction of bone thermal necrosis, all of the above tests were performed first under two cooling modes commonly used for bone drilling in operation rooms: without cooling and external cooling with normal saline. Many studies investigated the bone thermal necrosis merely under these two modes. To investigate the efficiency of gas coolants, the above tests were repeated for internal cooling mode with CO_2_ gas. CO_2_ was selected from among the applicable gas coolants for this study as this gas does not have any biocompatibility problem or infection risk according to previous studies and is suitable for using as coolant during bone drilling [18, 33, 34]. The use of CO_2_ is also very common in medicine, e.g. in laparoscopy operation. The conventional drilling machines, however, are not able to internally transfer CO_2_ gas through the body of drill bit directly to the point of drill bit and ultimately to the drilling site. On the other hand, manual drills are commonly used for bone drilling in operating rooms. Therefore, for better and more realistic simulation of operating room conditions, a manual drill with the ability of direct gas transfer to the drilling site was first developed in this study (Fig. 1a, b) 0.16 drill bits of type Mitsubishi Materials MVS0450X05S060 MVS Series Solid Carbide were used for bone drilling in tests (Fig. 1c). According to standard, each drill bit was used for drilling 40 holes and then was changed [15]. The rotational speed of the drill bit was measured by a digital tachometer (model: Victor DM6234P). The common method used in papers to assess thermal necrosis was to place a thermometer to measure the temperature at the bone drilling site. To this aim, a two-channel thermometer (model: TM-925 Lutron thermometer) was used for measuring the temperature at drilling site (Fig. 1d). The thermometer had a measurement range of − 50 to 1300 °C and an accuracy of 0.1 °C. It should be noted that the thermocouple was planted based on available protocols at a distance of 0.5 mm from the wall of central hole and at a depth of 3 mm for measuring the temperature at drilling site in all tests and then the time–temperature graph was registered by computer [9, 35]. The location of thermocouple planting was coated with a thermally conductive paste to minimize heat loss and raise precision.

Thermography was used to examine the thermal distribution on body surface or bone surface at drilling site [36]. An infrared camera (model: FLIR C2) was used for this purpose. The temperature range of the camera was − 10 to 150 °C, the thermography resolution 4800 pixels, the thermal sensitivity 0.1 °C and the spectral range 7.5–14 μm. The software used for thermography was FLIR TOOLS. The function of the infrared camera was based on radiation heat transfer and the governing relations were consistent with Stephen–Boltzmann’s relations. It should be noted that in the present study, the emissivity coefficient of bone was assumed to be 0.98 [37]. In the first stage of the experimental test, the infrared camera was placed on a special pod 50 cm away from the drilling site. The camera was then connected to a computer through a cable so that the operator could monitor the temperature throughout the process. In other runs, a higher resolution was needed to take photos from the drill bit following the drilling operation. For this purpose and to obtain better results, the camera was placed 25 cm away from the drilling site. Further to ensure the calibration of the thermography camera, the temperature at the bone surface was also measured by a Lutron thermometer and it was ascertained that the temperatures measured by the thermography camera and the thermometer are equal (with an error of less than 0.9%).

It is worth noting that in all figures, # mm means that the distance between lateral holes and the initial hole is # millimeters and # s means that the time delay between each drilling to the next drilling is # seconds. Furthermore, using “*w*” for time delay in figures means that there is no delay between each drilling to the next drilling and the drillings have been carried out without delay.

### Statistical analysis

Data registration was done after repeating each test seven times. Then, the coefficient of variation (CV) of all the data was calculated and the results of Table 1 showed that this parameter in all categories of data was less than 4.8.0%. After ensuring that the CV and the standard deviation (SD) of the data are within the allowable range, the mean temperature was calculated (Table 1). All the values used in the sections Results and Discussion are the means of these temperatures. It should be noted that mean, SD and CV of data were calculated with SPSS software version 22.0.

**Table 1:** Statistical analysis on the results of tests

Cooling mode	Distance (mm)/time delay (s)	Mean (°C)	Standard deviation	Coefficient of variation
Without cooling	6/0	61.4	2.4	3.9
	6/5	60.9	2.6	4.3
	6/10	60.4	2.7	4.5
	12/0	61.1	2.5	4.1
	12/5	59.6	2.8	4.7
	12/10	59.2	2.7	4.6
	16/0	60.6	2.4	4.0
	16/5	60	2.5	4.2
	16/10	59.6	2.6	4.4
Cooling with normal saline	6/0	84.2	3.2	3.8
	6/5	81.8	2.6	3.2
	6/10	78.6	2.8	3.6
	12/0	60.3	2.9	4.8
	12/5	57.8	2.6	4.5
	12/10	55.8	2.5	4.5
	16/0	46.9	2.2	4.7
	16/5	46.8	2.3	4.8
	16/10	46.5	2.3	4.8
Cooling with CO_2_	6/0	45.2	1.6	3.5
	6/5	45.1	1.4	3.1
	6/10	45.1	1.4	3.1
	12/0	45.1	1.4	3.1
	12/5	45	1.5	3.3
	12/10	45	1.6	3.6
	16/0	45	1.5	3.3
	16/5	44.9	1.4	3.1
	16/10	44.8	1.6	3.6

Mean is the mean of the maximum temperatures of 7 tests

## Results

### The effect of spatial distance between drillings

**Fig. 2:**
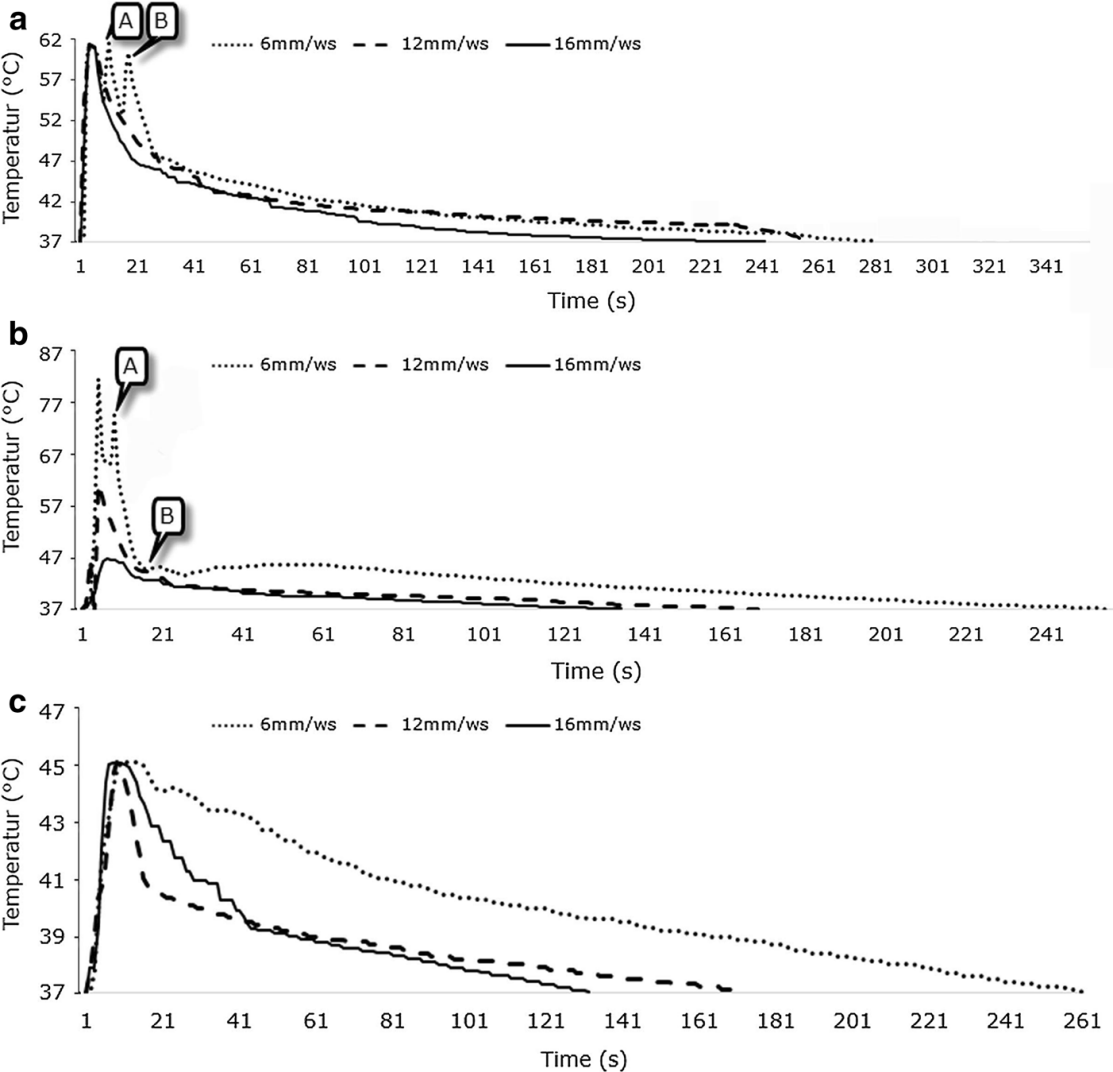
Graphs of temperature–time in initial hole during bone drilling **a** without cooling, **b** normal saline coolant and **c** CO_2_ coolant, while the distances are variable and the time delay is constant (without delay)

All investigations in this section were performed by changing the spatial distance between the centers of the laterals and initial holes from 6 to 16 mm while drillings were done without time delay. In drilling at a distance of 6 mm, the temperature–time graph of the drilling site of initial hole experienced temporal temperature increases from 54.1 to 61 °C and from 52.7 to 60.5 °C following drilling of lateral holes without cooling (Fig. 2a; points A and B). With increasing the distance from 6 to 16 mm, however, these thermal effects dropped sharply and thus it might be stated that the lateral drillings at distances of 12 and 16 mm, unlike distance of 6 mm, had no notable thermal effect on the initial hole (Fig. 2a). The maximum temperature in this cooling mode was 61.4 °C. In contrast to drilling without cooling, drilling the lateral holes using the external cooling mode with normal saline affected the trend of temperature–time graph only at the distance of 6 mm and only during the first lateral drilling (Fig. 2b). This phenomenon could be attributed to the effect of using normal saline as external coolant. The maximum temperature in this cooling mode had a completely different status at various distances (Fig. 2b). According to Fig. 2c, drilling the lateral holes at distances of 6, 12, and 16 mm had no specific effect on the temperature–time graph of initial hole when the internal cooling with CO_2_ gas was used. The maximum temperature during drilling with CO_2_ gas as coolant was 45.2 °C.

**Fig. 3:**
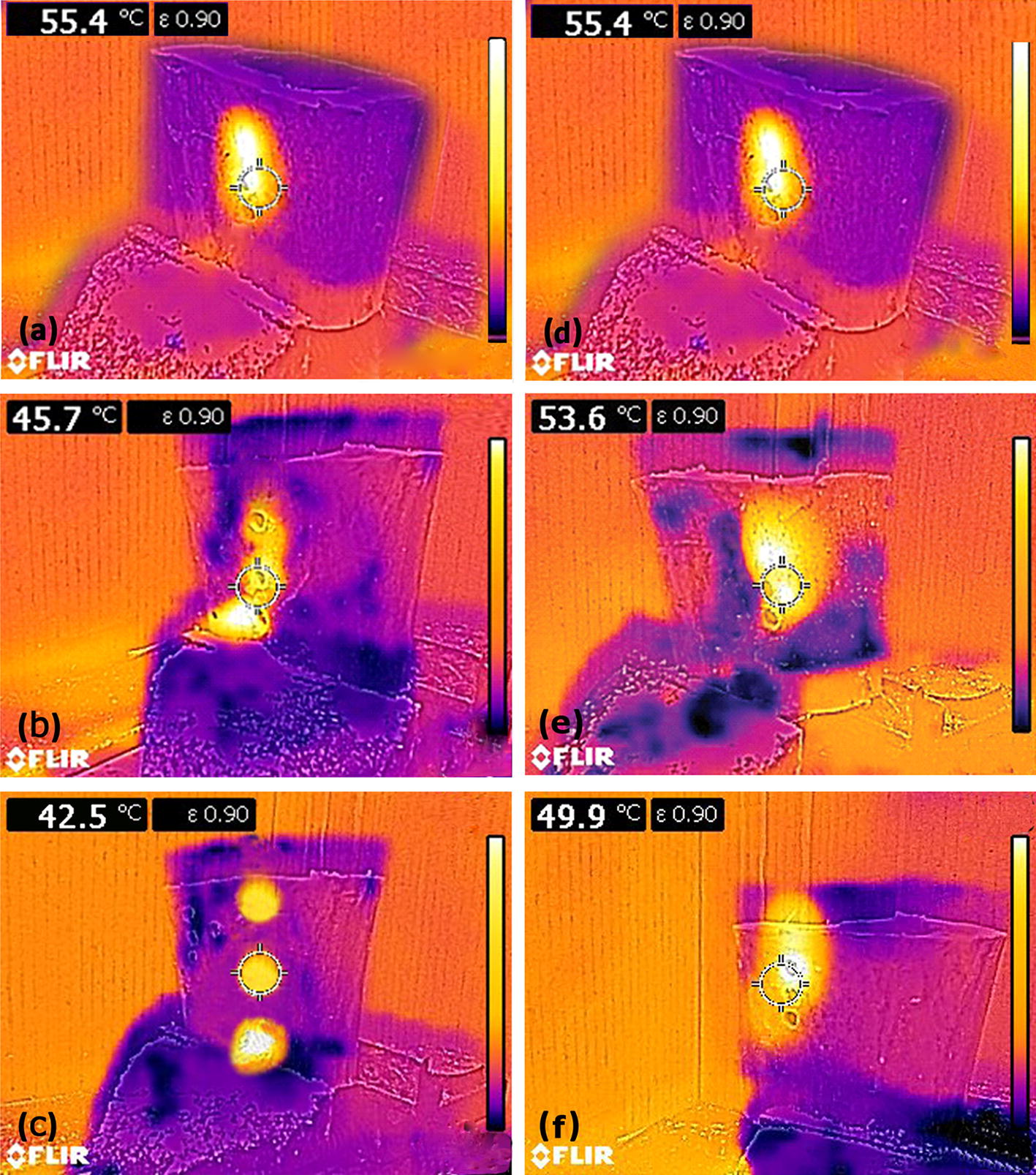
**a**–**c** Thermographic images of bone drilling without cooling and without delay. Distances between the holes in **a**–**c** are 6, 12 and 16 mm, respectively. **d**–**f** Thermographic images of bone drilling without cooling at a distance of 6 mm. Time delays between drillings in panel d, e and f are 0, 5 and 10 s, respectively

In assessment of bone thermal necrosis, examining the temperature distribution on the surface of drilling site is very important [30, 37, 38]. Figure 3a–c, which has been obtained from bone thermography, shows heat accumulation due to the effect of lateral drillings without cooling on the initial hole at the surface of drilling site. According to Fig. 3a–c, increasing the distance between the initial and lateral holes during drilling without cooling causes the heat accumulation at the surface of drilling site of the initial hole due to lateral drillings to decrease. With increasing the distance from 6 to 12 and 16 mm, the surface temperature decreases by 17.5% and 23.3%, respectively. The results also show that the temperature drop is, respectively, 8.3% and 14.8% during drilling with normal saline as coolant and 0.7% and 1.8% during drilling with CO_2_ gas as coolant.

### The effect of time delays between drillings

**Fig. 4:**
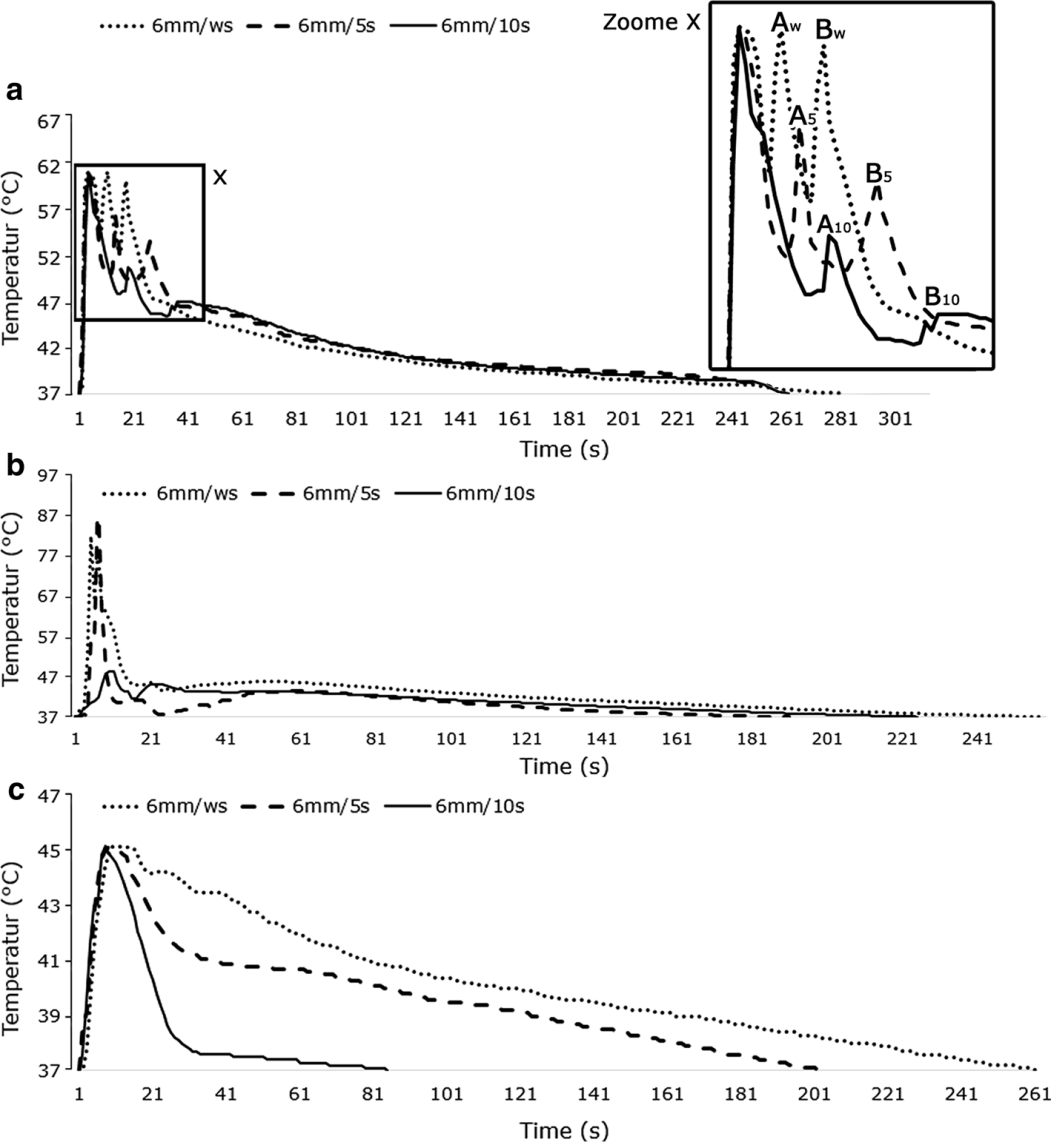
Graphs of temperature–time in initial hole during bone drilling **a** without cooling, **b** normal saline coolant and **c** CO_2_ coolant, while the time delays are variable and the distance is constant (6 mm)

All examinations in this section were performed by changing the time delay between the initial drilling and lateral drillings from zero to 10 s while the distance between the centers of the lateral holes and the initial hole was 6 mm. In the case of drilling without cooling and without delay, the lateral drillings led, respectively, to a temperature increase of 13.3% and 14.8% in the temperature–time graph of the initial hole (Fig. 4a; points *A*_w_ and *B*_w_). The temperature dropped after completion of each lateral drilling and the graph returned to its main trend. The relevant amounts were, respectively, 13.2% and 9.1% for drilling with a time delay of 5 s (Fig. 4a; points *A*_5_ and *B*_5_) and 6.0% and 3.2% for a time delay of 10 s (Fig. 4a; points *A*_10_ and *B*_10_). The maximum temperature at the drilling site in this cooling mode exceeded the threshold of human bone thermal necrosis (47 °C). According to Fig. 4b, c, however, lateral drillings while using the normal saline and CO_2_ gas as coolants caused no particular changes in the trend of the temperature–time graph of the initial hole. The maximum temperature in CO_2_ cooling mode did not exceed 47 °C.

Figure 3d–f shows heat accumulation at the surface of drilling site of the initial hole due to the effect of lateral drillings without delay. The maximum surface temperature for drilling without delay, with a delay of 5 s and with a delay of 10 s has been 55.4, 53.6 and 49.9 °C, respectively (Fig. 3d–f). Thus, by increasing the time delay of lateral drillings with respect to the initial hole drilling from zero to 5 s and 10 s, the heat accumulation in the initial hole has been moderated and the maximum surface temperature has been reduced by 3.2% and 9.9%, respectively. The results also show that the temperature drop has been, respectively, 1.3% and 5.3% while using normal saline as coolant and 0.5% and 2.1% while using CO_2_ coolant.

## Discussion

**Fig. 5:**
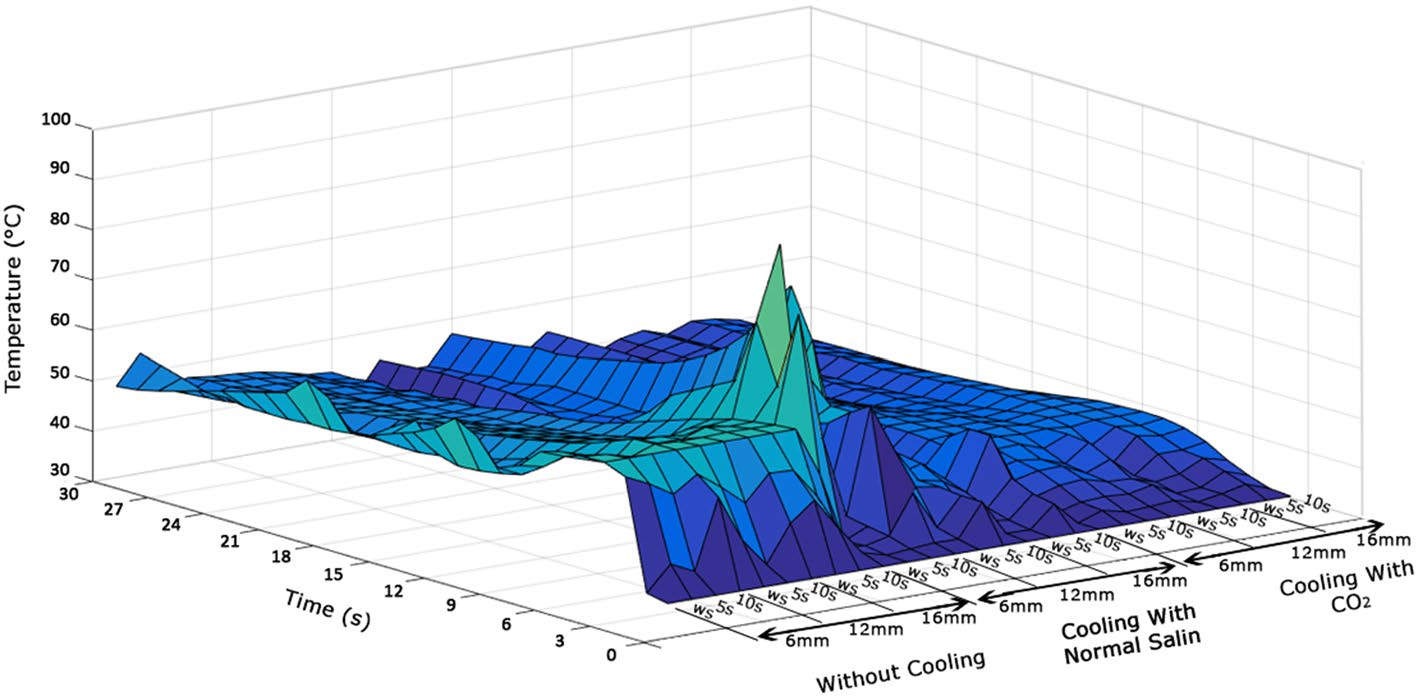
3D graph for comparison of trend of temperature–time graph under different time delays and distances

Several factors including the accumulation of chips, the force of chip formation and the friction between drill bit, hole wall and chips play a role in occurrence of bone thermal necrosis during drilling. In “Results” section, the effects of these factors are investigated in the framework of examining the trend of temperature–time graph, the maximum temperature at drilling site and the temperature distribution on the surface of drilling site. The results show that the effect of lateral drillings without cooling on the trend of temperature–time graph of initial hole is considerable at the distance of 6 mm (Fig. 2a) and even raising the time delay between drillings to 10 s cannot eliminate this effect at this distance (Fig. 4a). This effect existed in the external cooling with normal saline during drilling without delay and at the distance of 6 mm too. In internal cooling with CO_2_ gas, however, this effect was not considerable in any of the states. The results of Figs. 2 and 5 show that the maximum temperature of drilling site during drilling with CO_2_ coolant does not exceed the threshold of bone thermal necrosis (the maximum temperature has been 3.8% lower than the trend of temperature–time graph and threshold of thermal necrosis). Thus, drilling with CO_2_ coolant can be proper option regarding the maximum temperature at drilling site and the trend of temperature–time graph. The temperature at the surface of drilling site returned under the threshold value of bone thermal necrosis with increasing the distance from 6 to 12 mm in drilling without cooling (Fig. 3a–c) but it did not remain under the threshold temperature of thermal necrosis with decreasing the time delay from 10 to zero s (Fig. 3d–f). Furthermore, the lowest temperature of the surface of the drilling site at all distances and time delays was related to drilling with CO_2_ coolant.

**Fig. 6:**
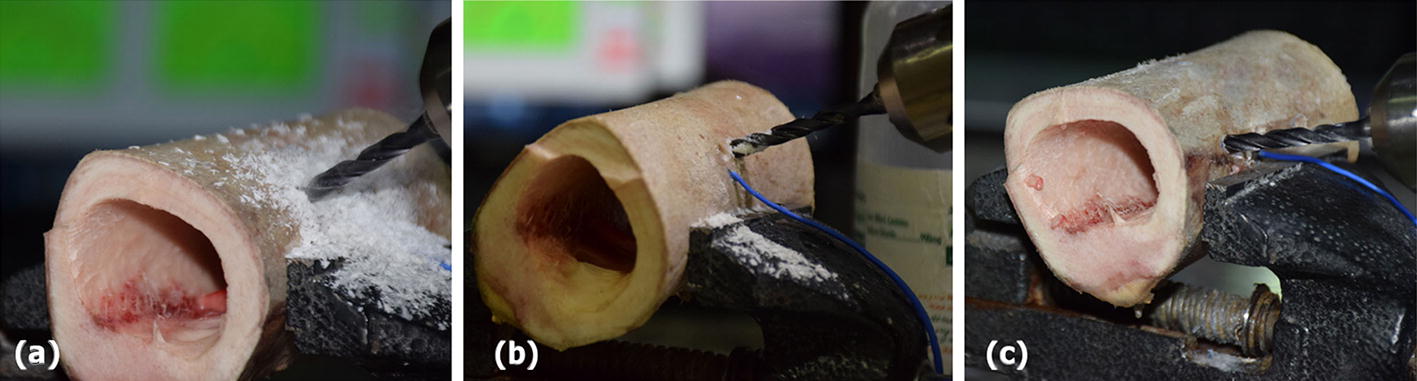
Chips of bone drilling with following cooling modes: **a** without cooling; **b** cooling with normal saline; **c** cooling with CO_2_

These results show that only the drilling with CO_2_ coolant can be the most suitable option for bone drilling regarding all the above-mentioned considerations (Fig. 5). One reason for this phenomenon can be the difference in chips removal during drilling using each of these three cooling modes. Due to lack of coolant in drilling without cooling, the chips move hardly out of the drill bit groove and this leads to accumulation of chips and increases in friction between the drill bit and chips and provides ultimately the conditions for undesirable parameters affecting bone thermal necrosis (Fig. 6a). During drilling with normal saline as coolant, the contact of chips with the coolant converts them into a sticky paste (Fig. 6b). This, especially following the rise of drilling depth, obstructs the drill bit groove and provides the conditions for chips accumulation, temperature confinement at the bottom of hole and increase in friction between the chips and the wall. In addition to above problems, drilling process using this cooling mode is unreliable and unstable as the cooling procedure is manual and non-uniform and there is no proper control over the cooling conditions. For example, in two drilling protocols using normal saline as coolant, first in drilling without time delay when the distance between the holes was 16 mm (Fig. 2b) and second in drilling with a time delay of 10 s and a distance of 6 mm (Fig. 4b), the maximum temperature at drilling site of the initial hole has been under the threshold of bone thermal necrosis. The reason of this is the manual cooling procedure and lack of uniformity of the cooling conditions with normal saline so that in some cases such as the two above-mentioned cases, spraying normal saline may be accidentally in a favorable manner which results in considerable reduction of the maximum temperature at the drilling site and in some other cases, the level of maximum temperature is even worse than that in drilling without cooling (Fig. 5).

It should be noted that even if, in contrary to the common practice of manual spray of normal saline coolant in operating rooms, this process is performed uniformly with automatic equipment, the use of normal saline as coolant is associated with the risk of infection at drilling site [35]. Further, normal saline, due to some limitations, cannot be used in some surgical procedures [19]. Therefore, drilling using normal saline coolant does not always yield desirable results as the examination of the parameters affecting the thermal necrosis.

In drilling with CO_2_ coolant, however, the injected gas is transferred directly from the body of drill bit to the point of drill bit and cools the bottom of hole quickly, and on the other hand, the pressure of the injected gas causes the chips to move faster out of the drill bit groove and prevents chips accumulation (Fig. 6c). All these parameters contribute to the most favorable drilling conditions using CO_2_ coolant.

**Fig. 7:**
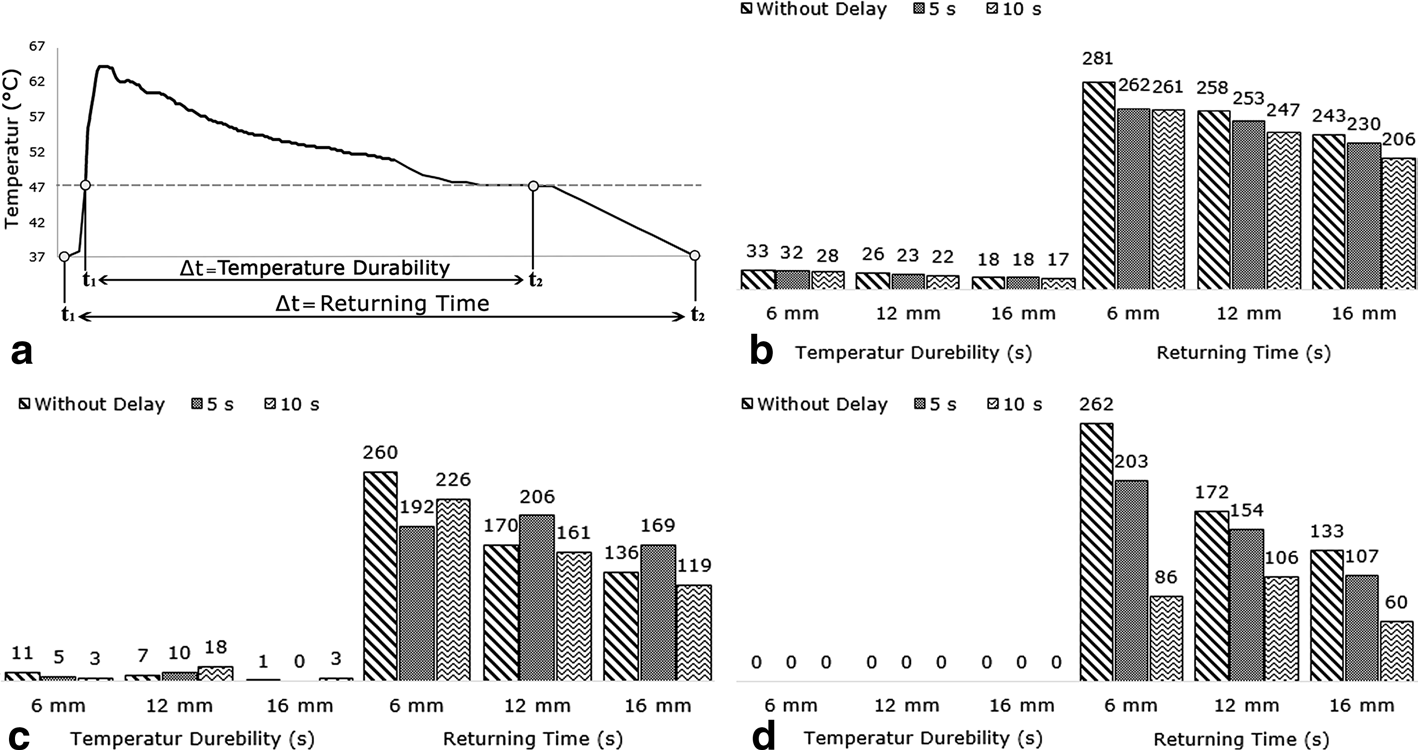
**a** Shows TD and RT in temperature–time graph and TD and RT graph at various distances and time delays: **b** without cooling, **c** with normal saline coolant, **d** with CO_2_ coolant

The important point is that the duration of exposure of bone cells to the extra-temperature generated during bone drilling should be investigated in addition to examination of previous parameters to assess the conditions of bone thermal necrosis completely. Thus, in addition to the above parameters, the temperature durability (TD), which indicates the durability of the drilling site temperature at a level above the 47 °C, and the returning time (RT), which indicates the time taken for return of the drilling site temperature to initial temperature (Fig. 7a), are extracted from Figs. 2 and 4 and examined. In drilling without cooling, increasing the time delay between drillings and increasing the distance between the holes reduced TD and RT according to Fig. 7b. Drilling with CO_2_ coolant had the same trend; of course, TD and RT were in this cooling mode smaller than those in drilling without cooling and conditions were more favorable (Fig. 7d). The most favorable conditions for the growth of bone cells were provided in drilling with CO_2_ coolant because the time of cells exposure in this cooling mode to a temperature above 47 °C (which results in death of bone cells and impaired bone formation) was zero (Fig. 7d). It should be noted that there was no significant relationship between the results of TD and RT in various distances and time delays during drilling with normal saline coolant. This was due to the manual cooling process and lack of control over drilling conditions (Fig. 7c). It is worth noting that the purpose of this study was to investigate and compare drillings in actual conditions of operation room and, therefore, no attempt was made to create an abnormal uniformity in spraying normal saline for achieving an abnormal homogeneity in results of drilling with this coolant. The results of this section also show that drilling with two common cooling modes, even in case of controlling distances between holes and time delays between drillings, will not yield favorable conditions. However, the risk of the incidence of bone thermal necrosis, even in drilling without delay or at the distance of 6 mm, disappears completely only when CO_2_ coolant is used.

Although numerous studies have been conducted in the past on the reduction of thermal necrosis and improvement of drilling conditions in bone, the results of this study can be helpful for controlling the distance between holes and time delays between drillings. As the surgery speed is important in many types of emergency orthopedic surgeries, the results of this study can alleviate this concern. These results show that using CO_2_ as coolant during bone drilling keeps thermal necrosis and thermal accumulation within the allowable range even at limited distances and time delays. Furthermore, the mechanical strength is mainly considered in designing the distances between holes in implants and plates [39–41] and less attention is paid to the conditions of thermal necrosis while designing the locations of holes. The results of this study can also be useful for eliminating this defect. Many studies evaluated some biomechanical parameters using computer simulation [42–50]. Hence, in future researches are suggested that computer simulations be used to evaluate the trust force and thermal stress of the subject under study in the present research.

## Conclusions

The results of examinations of the trend of temperature–time graphs, the maximum temperature at the drilling site, the temperature distribution on the surface of the drilling site, TD and RT show that using CO_2_ coolant, compared to two commonly used cooling modes, even in cases that the distances between holes in implants or plates are small (e.g. 6 mm) and even without any time delay between drillings, eliminates the risk of occurrence of thermal necrosis and potential post-operation complications completely. The results of this study can be useful for controlling the distances between holes and time delays between drillings required for bone fixation in orthopedic surgeries, especially in emergency orthopedic surgeries. The manufacturers of plates and implants can also use these results for designing the location of holes in implants and plates.

## Data Availability

Not applicable

## Abbreviations

SD: standard deviation
CV: coefficient of variation
TD: temperature durability
RT: returning time
